# Feasibility and outcomes of a real-world regional lung cancer prehabilitation programme in the UK

**DOI:** 10.1016/j.bja.2022.05.034

**Published:** 2022-07-13

**Authors:** Patrick Bradley, Zoe Merchant, Kirsty Rowlinson-Groves, Marcus Taylor, John Moore, Matthew Evison

**Affiliations:** 1 Manchester Thoracic Oncology Centre, Wythenshawe Hospital, Manchester University NHS Foundation Trust, Manchester, UK; 2 Division of Infection, Immunity and Respiratory Medicine, University of Manchester, Manchester, UK; 3 Greater Manchester Cancer Prehab4Cancer and Recovery Programme, Manchester, UK; 4 Department of Thoracic Surgery, Manchester University NHS Foundation Trust, Wythenshawe Hospital, Manchester, UK; 5 Division for Anaesthesia, Peri-Operative Medicine and Critical Care Services, Manchester Royal Infirmary, Manchester University NHS Foundation Trust, Manchester, UK

**Keywords:** exercise therapy, lung cancer, prehabilitation, quality of healthcare, thoracic surgery

## Abstract

**Background:**

Prehabilitation, or multimodality patient optimisation before major treatment, has demonstrated meaningful improvements in patients' outcomes. In the setting of lung cancer surgery, postoperative complications and length of hospital stay are reduced, but there is currently limited access to prehabilitation. Prehab4Cancer (P4C) is an innovative regional programme serving all areas of Greater Manchester (GM).

**Methods:**

The lung cancer P4C service commenced in 2019 as a collaboration between the GM Cancer alliance and 12 leisure and community organisations. Patients planning surgical resection could be referred to receive exercise, nutrition, and well-being assessment and interventions before surgery. We evaluated the programme's feasibility, uptake, and outcomes during the 11 months before COVID-19 restrictions.

**Results:**

In total, 377 patients were referred to the lung cancer P4C service from all 11 hospitals in GM. Of the patients reached by telephone, 80.0% (*n*=280/348) attended initial P4C assessment, which occurred a median of 8 days (inter-quartile range [IQR]: 4–14) after referral. In addition, 74.3% (*n*=280/377) attended for baseline assessment and 47.7% (*n*=180/377) completed prehabilitation, attending a median of six sessions (IQR: 4–9). Statistically significant improvements in all objective physiological and subjective functional assessments were observed preoperatively, including a mean increase in the incremental shuttle walk test of 50 m (95% confidence interval: 25–74; *P*<0.001).

**Conclusions:**

The P4C programme demonstrated feasibility at scale, high uptake, and promising impact on the status of patients with lung cancer before surgery. P4C is the first regional prehabilitation service internationally, and this evaluation provides a framework for implementing similar services in other regions.

**Editor's key points**

• Postoperative complications and length of hospital stay are reduced by prehabilitation.
• Prehab4Cancer is an innovative regional programme serving the Greater Manchester area in the UK, in which patients planning surgical resection receive assessment and interventions before surgery.
• This paper describes the programme's feasibility, uptake, and outcomes in 377 patients referred before the COVID-19 pandemic.
• The programme was feasible at scale with high uptake and had a positive impact on preoperative physiological and subjective functional assessments, providing a framework for wider implementation.

Lung cancer is the most common cause of cancer-related death in the UK with approximately 35 000 deaths every year.^1^ Surgical resection at an early stage offers the best chance of long-term survival, and the number of lung cancer operations in the UK is increasing year on year (∼7000 per year currently).^2^ Further improvements in early detection, such as through targeted screening, are leading to a greater proportion of patients with lung cancer being diagnosed at a stage where surgical resection is possible.^3^^,^^4^ Therefore, there is renewed focus on optimising outcomes in lung cancer surgery.

Prehabilitation describes patient optimisation before treatment, such as lung cancer surgery.^5^ Exercise training is a core intervention, but prehabilitation also involves allied components, such as nutritional and psychological well-being assessment and support. It is increasingly recognised as an important phase of cancer treatment pathways, reducing complication rates, improving functional capacity, and improving quality of life.^6^^,^^7^ A recent meta-analysis of RCTs of exercise training before lung cancer surgery demonstrated a significant reduction in the rate of postoperative complications (risk ratio 0.42; 95% confidence interval [CI]: 0.25–0.69), postoperative length of stay in hospital (mean difference –2.29 days; 95% CI: –0.98 to –3.59), and improved exercise capacity (6 min walk distance mean difference +37.6 m; 95% CI: +20.5 to +54.7).^8^ Given this strong evidence supporting the efficacy of prehabilitation before lung cancer surgery alongside the increasing volume of lung cancer surgery, service delivery is the primary challenge.

Despite this evidence and its recommendation in international guidelines,^9^ there is a wide variation in prehabilitation provision across cancer services, rendering it unavailable to a large proportion of patients. Whilst a small number of individual hospital-based services exist,^10^, ^11^, ^12^ implementation of resilient, sustainable, and effective prehabilitation services at scale across large geographical areas is a key priority. This challenge is not unique to the UK; a recent survey of thoracic surgeons in Australia identified a high perceived need for prehabilitation, but only 16.7% of respondents could access services.^13^

The Greater Manchester (GM) Prehab4Cancer (P4C) programme is a system-wide prehabilitation programme for patients in GM delivered as a collaboration between hospital-based clinical teams, the regional cancer alliance (GM Cancer), and the community leisure sector (GM Active). Here, we examine the feasibility, uptake, participation, and clinical outcomes from this service delivery model.

## Methods

### Service setting

GM is a metropolitan county in the Northwest of England with a population of 3.2 million, with ∼2500 patients diagnosed with lung cancer annually across the GM conurbation. There are 11 acute NHS hospitals in GM. Thoracic surgery is provided at a single site. The cancer system is led by the ‘GM Cancer’ alliance, which sets the cancer priorities for the region and allocates transformation funding aligned to these priorities. ‘GM Active’ is a collective of 12 leisure and community organisations from across GM, with a shared vision to get more people physically active.^14^ This collaboration comprises 87 leisure and sports facilities across the region, ensuring there is a facility within 5 miles of all GM residents. GM active is supported through numerous partnerships, including the local health authorities, the GM Health and Social Care Partnership, the GM Combined Authority, GreaterSport, UKActive, and Sport England.

### P4C inception

The series of events that culminated in the P4C programme has been published.^15^ In short, a team of GM perioperative clinicians delivering an enhanced surgery programme ERAS+ (Enhanced Recovery After Surgery Plus^16^) formed a partnership with GM Active to develop and deliver a GM-wide community-based multimodal prehabilitation programme for patients preparing for cancer surgery. This was supported by transformation funding from the GM Cancer Alliance, which had made prehabilitation and rehabilitation implementation a regional cancer priority. The P4C programme was allocated £1.3 million over 2 yr to support 2000 patients through a prehab–rehab programme. This funding allowed recruitment of a P4C team consisting of a clinical lead, a transformation programme lead, a primary care lead, an operational programme manager and a team of six exercise specialists (Level 4 cancer rehabilitation qualified exercise practitioners able to design, agree, and adapt a physical activity programme to aide patients living with cancer), three Level 3 qualified exercise instructors (Level 3 qualification is the standard to practice as a personal trainer), and a referral coordinator deployed within the GM Active system. The P4C team engaged with each cancer pathway board included in this initial project (lung, colorectal, oesophago-gastric) to develop site-specific referral pathways and engage with the local teams at all GM hospitals *via* a site-specific P4C subgroup. The P4C Lung subgroup was established in September 2018 with a planned service launch date of April 2019. The Lung subgroup included a multidisciplinary team (MDT) of healthcare professionals, patient representatives, and P4C delivery team members. The subgroup agreed and defined the prehabilitation pathway and the red–amber–green (RAG) ratings for key performance indicators (KPIs), defined *a priori*, as set out as follows.

### Lung cancer P4C pathway

In the pilot programme, patients with lung cancer were eligible for P4C if surgical resection was planned, as it is this cohort of patients who have the clearest evidence of benefit.^8^ The inclusion criteria were lung cancer MDT-agreed diagnosis of primary lung cancer with a treatment recommendation of surgical resection, aged 18 yr or over, registered with a GM primary care service, able to access the programme either independently or with support from a carer/family member, indicated informed consent to be referred, and walked more than 250 m on the incremental shuttle walk test (ISWT). As a community programme without clinical facility support, embedded risk assessment at all stages of the pathway was crucial to mitigate the risk of adverse events during prehabilitation. The ISWT is widely used across GM in the lung cancer pathway because of its reproducibility, accessibility, and a strong evidence base.^17^ An ISWT >40 shuttles (400 m) correlates to a VO_2max_ >15 ml kg^−1^ min^−1^ on cardiopulmonary exercise testing (CPET), a value deemed to represent good physiological function in national guidelines on the risk assessment for lung cancer surgery.^18^^,^^19^ However, the shuttle walk may underestimate VO_2max_ at the lower ranges with more than half of patients with a shuttle walk <250 m having a VO_2max_ >15 ml kg^−1^ min^−1^.^20^^,^^21^ More recent data demonstrated that a shuttle walk of >25 shuttles (250 m) has a 90% positive predictive value for VO_2max_ >15 ml kg^−1^ min^−1^.^22^ Consequently, the P4C Lung subgroup recommended that patients with an ISWT >400 m would be suitable for a universal (unsupervised) prehabilitation programme, those with an ISWT of 250–400 m would be suitable for a targeted (supervised) prehabilitation programme, but those with an ISWT <250 m would require further assessment with CPET. After CPET, patients with a VO2_max_ >15 ml kg^−1^ min^−1^ were deemed eligible for a universal (unsupervised) prehabilitation programme, those with a VO_2max_ 10–15 ml kg^−1^ min^−1^ would be suitable for a targeted (supervised) prehabilitation programme, but those with a VO_2max_ <10 ml kg^−1^ min^−1^ generally signified prohibitive risk for lung cancer surgery and were also unlikely to be able to safely complete a community-based exercise programme. These patients were therefore deemed ineligible for the programme. A future requirement for cancer prehabilitation will be the development of specialist pathways for patients with greater levels of frailty and comorbidity to ensure equity of access to the benefits of prehabilitation safely, but this was not available in this transformation and implementation phase.

Eligible patients were identified at the lung cancer MDT and were provided with written information on the programme and on the benefits of prehabilitation. Education was provided to clinicians about the programme and strategies to communicate these benefits to patients. Referral to P4C was performed using an online referral portal. Patients were initially contacted by telephone to organise a face-to-face appointment at one of 17 first assessment clinics. At this assessment, medical history and baseline assessments were performed and an individualised prehabilitation programme prescribed. The patient could then complete this programme at any one of the 87 GM Active leisure facilities. The same functional and quality-of-life assessments were repeated immediately before the date of surgery. After treatment, a 12 week postoperative rehabilitation programme was provided.

### Prehabilitation intervention

Patients were offered a prehabilitation programme tailored to their baseline fitness, as determined at assessment clinic. Patients were triaged into ‘universal’ or ‘targeted’ pathways, based on principles of NHS England's Personalised Care model.^23^ Exercise prescriptions for the targeted pathway included three supervised group gym sessions per week. For the universal pathway, patients could exercise independently with weekly monitoring with the exercise specialist. Exercise prescriptions included reduced-exertion high-intensity interval training and resistance training prescribed according to percentage of maximum HR or perceived rate of exertion.^24^ Training prescriptions were escalated as fitness improved. Nutritional status was assessed at baseline and at intervals through the programme. Three risk categories were used to identify those in need of nutritional support, and each category received simple interventions or onward referral when required (Table 1). Psychological well-being is the third component of P4C, aiming to improve motivation, resilience, and quality of life through the period of distress that a new diagnosis of cancer brings. This was similarly assessed using a three-tier risk assessment mapped to interventions that P4C can provide (Table 1).

**Table 1:**
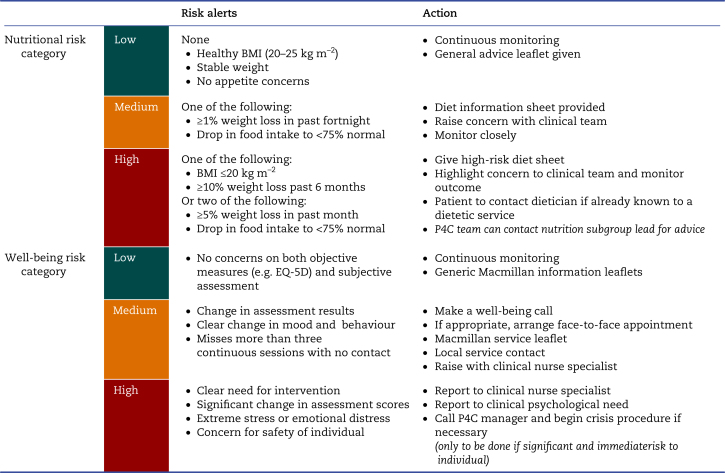
Prehab4Cancer nutritional and well-being support assessment and management frameworks. EQ-5D, European Quality of Life Five Dimensions; P4C, Prehab4Cancer.

### Study period

This evaluation of the Lung P4C programme describes the period from the service launch in April 2019 until the suspension of face-to-face services because of the COVID-19 pandemic in March 2020 (11 month period).

### Measures of feasibility, uptake, participation, and outcomes

We hypothesised that offering P4C to patients with lung cancer as a standard of care would be feasible at regional scale, have good uptake and participation, and have a positive impact on clinical outcomes and quality of life. Feasibility would be judged by the engagement of clinical teams across the region to refer eligible patients *via* the online portal and for referrals to be actioned within the KPIs. With an estimated 500 lung cancer resections in GM per year (equivalent to ∼460 in this 11 month study period), we estimated that a referral rate of 75% (*n*=345) would represent a green RAG rating for feasibility (Table 2). To test uptake amongst referred patients, we calculated the proportion of those who were successfully contacted by phone (green RAG rating of >90%) and the proportion of those who completed an assessment (green >75%). The number of patients completing an assessment was also considered as a proportion of the total number of referrals on an intention-to-treat (ITT) basis (green >65% based on expected dropout at each step). To assess the feasibility of providing rapid access to prehabilitation to ensure progress through the cancer pathway, a KPI of 7 days or less from referral to first assessment clinic was established. To assess feasibility of the eligibility criteria and clinician selection for P4C, the proportion of patients deemed ineligible and unsafe to proceed with community prehabilitation at the first telephone call or when seen at the first assessment clinic was calculated (green <10%). To assess participation, we defined engagement with the programme as attending at least the initial face-to-face assessment session and any subsequent prehabilitation sessions up to point of surgery (green >75% of those attending the first assessment and >50% of the ITT population).

**Table 2:**
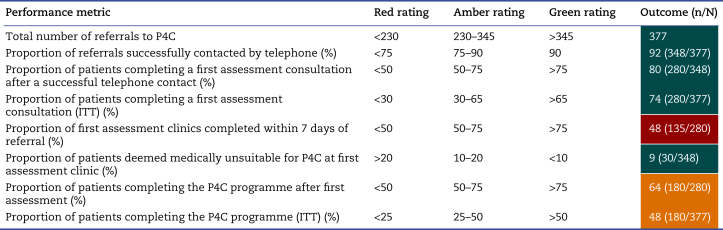
Feasibility and uptake red–amber–green rating definitions and performance for the Greater Manchester Prehab4Cancer programme, March 2019–April 2020. ITT, intention to treat; P4C, Prehab4Cancer.

Clinical impact and outcomes were explored using measures recorded during initial assessment and at the repeat assessment immediately before surgery. The following objective assessments were made at baseline and end of prehabilitation to assess fitness: ISWT, 6 min walk test (6MWT), 60 s sit-to-stand test (STS), hand grip dynamometry (HGD), BMI, and clinical frailty scale. Subjective measures were used to assess health-related quality of life at baseline and at follow-up: 12-item WHO Disability Assessment Schedule (WHODAS) 2.0, Self-Efficacy for Exercise (SEE) scale, International Physical Activity Questionnaire (IPAQ), and European Quality of Life Five Dimensions (EQ-5D) scores. Descriptive statistics were used to assess changes in these objective and subjective measures between the beginning and end of prehabilitation. Pearson's χ^2^ test was used for comparisons of categorical variables (IPAQ). For continuous variables, paired *t*-test was used to compare repeated measures of parametric data (6MWT, ISWT, STS, and HGD) and Wilcoxon signed-rank test for non-parametric data (WHODAS, SEE, and EQ-5D-5L).

## Results

### Feasibility, uptake, and participation

During the 11 month period evaluated, 377 patients were referred to the Lung P4C service, originating from 11 hospitals across the GM region (range: from nine to 70 referrals per centre). Amongst the 377 patients referred to P4C, 52.3% (*n*=197) were female, and the median age was 72 yr (inter-quartile range [IQR]: 66–77). Twenty-nine patients could not be contacted by telephone, primarily because the referrals did not include a valid telephone number. From the contacted patients (*n*=348), 80.5% (*n*=280) attended the first assessment. On ITT analysis, 74.3% (*n*=280/377) of patients referred completed an assessment. The median interval between referral and initial assessment was 8 days (IQR: 4–14) with 48.2% (*n*=135/280) of assessments within 7 days.

**Fig 1:**
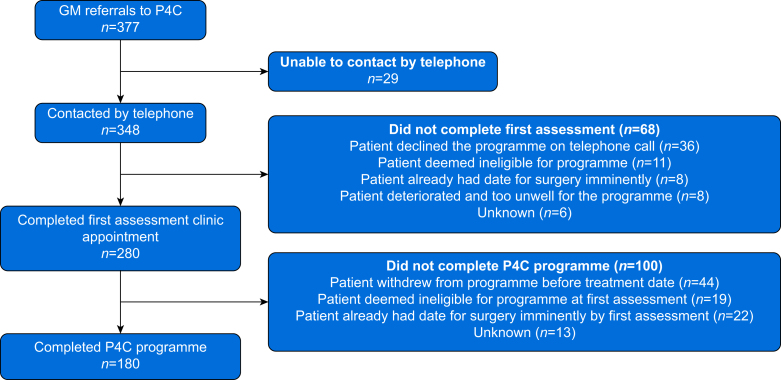
Flow diagram to illustrate uptake and participation rates at each stage of the Prehab4Cancer lung pathway. GM, Greater Manchester; P4C, Prehab4Cancer.

During the initial telephone contact and first assessment, 8.6% (*n*=30/348) were deemed medically unsuitable to participate in P4C. Overall, 64.3% (*n*=180/280) of patients who attended a first assessment went on to complete the prehabilitation phase with a median number of sessions completed of 6 (IQR: 4–9). The median interval from assessment to surgery was 36 days (IQR: 22–55). Overall participation on an ITT basis was 47.7% (*n*=180/377). Fig. 1 provides the reasons for non-participation at each stage of the pathway. Overall, 21.2% (*n*=80/377) of referred patients declined or withdrew. From the 377 referrals, 30 patients (8%) were deemed unsafe to participate or proceed with the programme because of concerns about their physiological reserve and safety to undertake the prescribed prehabilitation programme. During this service delivery period, there were no adverse events during participation in exercise sessions reported by the exercise specialists to the P4C steering group.

### Outcomes

**Fig 2:**
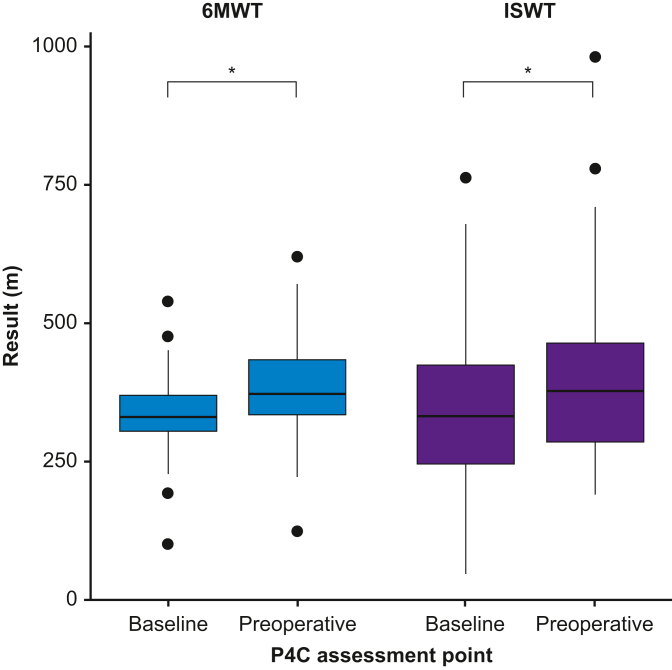
Comparison of patient-level changes in performance in 6 min walk test (6MWT) and incremental shuttle walk test (ISWT) before and after prehabilitation. ∗*P*<0.001; paired *t*-test. P4C, Prehab4Cancer.

**Fig 3:**
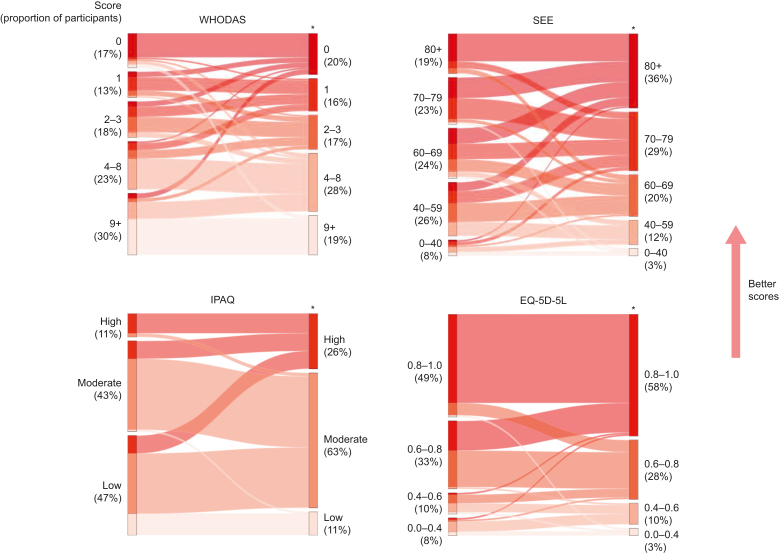
Comparison of changes in subjective assessment scores before and after prehabilitation. Proportions of participants in each score category are given in parentheses as the percentage of the cohort with repeated measures available (*n*=120). ∗*P*<0.001 for test of difference; Wilcoxon signed-rank test (WHO Disability Assessment Schedule [WHODAS], Self-Efficacy for Exercise scale [SEE], and European Quality of Life Five Dimensions [EQ-5D-5L]); Pearson's χ^2^ test (International Physical Activity Questionnaire [IPAQ]).

The results of objective and subjective assessments performed by P4C participants, including both initial assessment and at the end of prehabilitation (preoperatively), are shown in Table 3. Statistically significant improvements were observed in ISWT, 6MWT, STS, HGD, WHODAS, SEE, IPAQ, and EQ-5D. The mean difference in ISWT was +50 m (95% CI: +25 to +74; *P*<0.001). Amongst those participants with repeated subjective functional assessment results available, 30% (*n*=36/120) reported most disability (WHODAS score ≥9) at baseline, a proportion that reduced to 19% after prehabilitation. The proportion scoring best (WHODAS 0) improved from 17% to 20% preoperatively. Further detail regarding patient-level changes is shown in Fig 2, Fig 3.

**Table 3:** Physiological and functional assessments performed on Prehab4Cancer participants after referral to prehabilitation, and at the end of prehabilitation (before surgery), with differences calculated for those with repeated measures available. ^†^Mean (standard deviation); median (inter-quartile range); *n* (%). ^‡^Paired *t*-test (parametric repeated measures); Wilcoxon signed-rank test (non-parametric repeated measures); Pearson's χ^2^ test (categorical data). CI, confidence interval; HGD, hand grip dynamometry; IPAQ, International Physical Activity Questionnaire; ISWT, incremental shuttle walk test; SEE, Self-Efficacy for Exercise scale; STS, sit-to-stand test; WHODAS, WHO Disability Assessment Schedule 2.0; 6MWT, 6 min walk test.

Variable	*n*	Initial assessment^†^	End of prehabilitation^†^	*n* paired results	Difference (95% CI^‡^)	*P*-value^‡^
6MWT (m)	108	297 (98)	356 (114)	50	+43 (+31 to +54)	<0.001
ISWT (m)	145	361 (168)	405 (159)	56	+50 (+25 to +74)	<0.001
STS (repetitions in 60 s)	149	18 (8)	23 (8)	70	+4.8 (+3.5 to +6.0)	<0.001
HGD (kg)	265	26 (9)	26 (8)	105	+0.7 (+0.2 to +1.2)	0.011
WHODAS	280	5 (2–10)	3 (1–7)	120	–1.8 (–2.6 to –0.9)	<0.001
SEE	280	66 (49–77)	74 (63–81)	120	+7.1 (+4.4 to +9.9)	<0.001
IPAQ, *n* (%)	280			120		<0.001
Low		158 (56)	13 (11)			
Moderate		91 (32)	76 (63)			
High		31 (11)	31 (26)			
EQ-5D-5L	280	0.80 (0.68–0.88)	0.84 (0.71–1.00)	120		<0.001

## Discussion

To our knowledge, this is the first regional lung cancer prehabilitation service internationally to be offered as a standard of care and delivered to patients in the community *via* an established leisure network. The high number of referrals over an 11 month period (*n*=377 in a region with 458 lung cancer resections over the same time period) suggests good engagement by hospital-based clinical teams. Our rates of uptake and participation are comparable with those reported in clinical trials as a proportion of patients screened reaching inclusion in final analyses (range: 41–80%).^7^^,^^25^, ^26^, ^27^, ^28^

The referral pathway and service delivery model appear feasible, achieving ‘green’ rating on five out of eight predefined feasibility indicators. The programme scored ‘red’ for one indicator with only 48% of first clinic appointments being completed within 7 days of referral. However, the median time was 8 days, suggesting the service is close to achieving this indicator. The number of sessions attended provides some assurance that the programme delivers adequate prehabilitation before treatment. The low rate of patients deemed unsafe to proceed with the prehabilitation programme after referral and the lack of adverse events reported through the governance system provides evidence of appropriate patient selection using the ISWT and CPET parameters and assurance on the safety of the programme.

The objective measures of functional capacity and quality of life also provide assurance of effectiveness. The most recent meta-analysis of exercise training before lung cancer surgery, which demonstrated a reduction in postoperative complications of ∼50%, also demonstrated a mean increase in 6MWT of 37 m. We report mean increases in 6MWT and ISWT of 43 and 50 m, respectively. Hence, it can be inferred that a similar reduction in the rate of postoperative complications may also be achievable.

Quality of life is an understudied outcome measure in prehabilitation. This evaluation provides a comprehensive assessment of quality of life demonstrating improvements across multiple assessment scales, adding a further dimension to the benefits of prehabilitation.

One in five patients referred to the programme opted not to participate at different stages of the pathway. Reasons for non-participation are poorly understood. Self-reported reasons for participation in P4C include perception of the benefits of prehabilitation, which are influenced by the treating medical team. The implicit psychosocial support of having an element of coaching through a difficult patient journey is also thought to be important (unpublished data).

This service evaluation describes a regional standard-of-care intervention with no allocated control group. For this reason, the likelihood of selection bias was considered too significant to allow comparison of clinical outcomes, such as complication rates and early mortality between patients undergoing surgery who participated in P4C and those who did not. Participation data are incomplete owing to a lack of data on unsupervised exercise; reported participation rates are therefore an underestimate. Obtaining full ‘end-of-prehabilitation’ assessment data (*n*=120 completed) proved challenging, particularly given that scheduling such an appointment in advance of the surgery date, once known, requires significant service flexibility and patient availability. A strength of this evaluation lies in its potential to inform expansion of prehabilitation services. The P4C framework of a system-wide collaboration across clinical groups, the community leisure sector, and the regional cancer alliance is novel and could be adopted in other regions. The described RAG-rated indicators of feasibility, uptake, and participation could be used to benchmark other services and build a wider understanding of uptake and participation and strategies for optimising them.

Reflecting on the P4C programme journey in GM, there were some key pillars of success and some key challenges to overcome, relevant to all areas considering such programmes. The GM Cancer alliance must be praised for placing prehabilitation as one of the top cancer priorities for the region and investing one of its largest single cancer transformation funding awards to this programme. This transformation funding ensured adequately resourced clinical leadership and programme management, which were critical to success in the implementation phase. The programme deployed a strong governance structure from the outset with patient representation at every level (Supplementary Fig. S1). This ensured good engagement and communication across the cancer system, rapid development of agreed protocols/pathways, and regular quality assurance review from the moment of launch. The key strength of this programme is the collaboration with an existing community leisure service infrastructure and creating smooth referral pathways from NHS care to community leisure teams. Strong connections were made between clinical teams and the P4C exercise specialists, with healthcare professionals providing a regular programme of education for the P4C team in all aspects of cancer care to support their professional development and enhance the support they provided to patients with cancer.

One challenge was to embed a clinician-led discussion on the benefits of P4C within NHS consultations and ensuring that P4C referral became a standard of care. Referral rates did vary across different hospitals with some engaging more than others. To support this process, the P4C programme became a regular agenda item on the regional lung cancer board meetings where there was representation from all hospitals. Patient testimonials and outcome data proved to be valuable tools to increase referrals. Patients and family members/carers were signposted to easy-to-use, codesigned patient information leaflets and resources, such as the programme website (www.prehab4cancer.co.uk).^29^ Lung cancer teams were encouraged to record P4C referral within the treatment recommendations in lung cancer multidisciplinary team meetings to act as a reminder to the clinical staff in subsequent consultations. The service also benefited from utilising two opportunities to refer a patient to P4C within the lung cancer pathway: referral from the local hospital team at the time of diagnosis and on receipt of a referral to or clinical consultation at the regional thoracic surgery centre. An opposite issue encountered was some clinical teams referring patients too early in the pathway before completing their staging investigations and before MDT confirmation of the management plan. This sometimes led to difficult scenarios for the exercise specialists, going beyond their boundaries of practice, where patients would be asking them for test results and treatment plans, which risks compromising the relationship and trust building required between specialists and patients in a prehabilitation programme. This was addressed through communications *via* the lung cancer pathway board to ensure a standardised point of referral when the diagnosis, stage, and management plan (surgery) had been confirmed at a lung cancer MDT.

When the COVID-19 pandemic started in March 2020, P4C converted to a remote model of service delivery through telephone and video consultations, online sessions, and provision of simple home exercise equipment.^30^ This alternative service delivery model will be similarly evaluated for comparison. From October 2020, P4C expanded to include patients undergoing non-surgical curative-intent treatment. This is an understudied area where outcomes also ought to be evaluated.

## Conclusions

P4C has implemented a comprehensive prehabilitation service for patients with lung cancer across the GM region, demonstrating feasibility as a standard-of-care service at scale with appropriate levels of uptake and participation to ensure the meaningful clinical benefits already proved in RCTs. Measures of functional performance and quality of life improved amongst participants between initial and preoperative assessments. P4C provides a potential framework for further roll-out across large geographical areas and provides a standardised assessment of uptake, participation, and outcomes against which real-world services can be benchmarked.

## Authors' contributions

Study conceptualisation: ME, JM

Data collection/cleaning: PB, ZM, KR-G

Data analysis: PB, MT

Drafting of article: PB, MT

Review/editing of article: all authors
