# Supplementary material for: Feasibility and outcomes of a real-world regional lung cancer prehabilitation programme in the UK
Source: Br J Anaesth. 2022 Jul 13;130(1):e47–55. doi: 10.1016/j.bja.2022.05.034 (PMC9875904; doi:10.1016/j.bja.2022.05.034)

*Feasibility, uptake, participation and outcomes of a real-world regional lung cancer prehabilitation programme*

**Supplementary appendix**

**Figure S1**: Governance structure for the Prehab4Cancer programme.


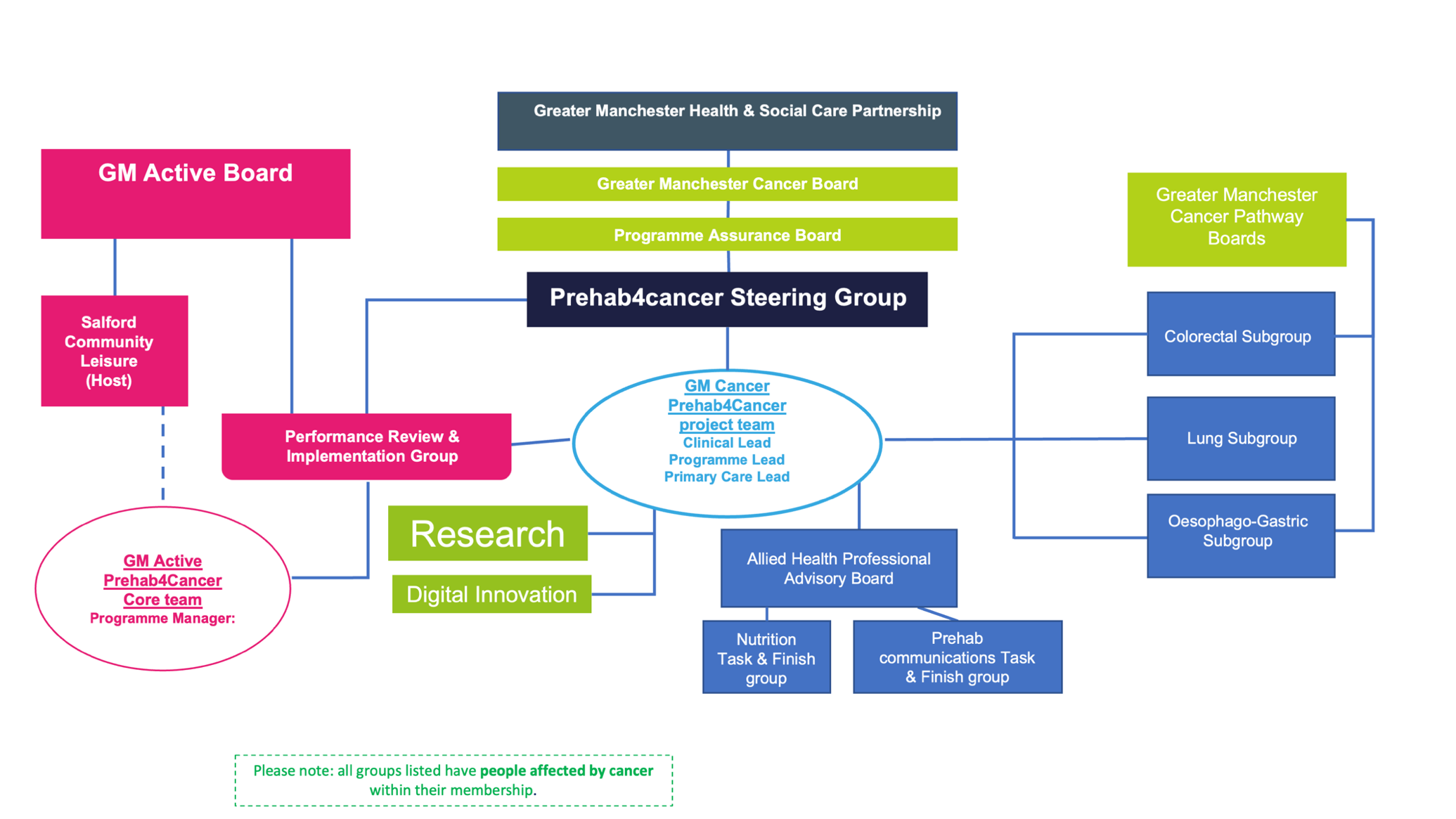

Supplement: Multimedia component 1 [file mmc1.docx]
